# Compartmentalization of High Infratemporal Fossa in Oral Cavity Squamous Cell Carcinomas and Its Impact on Clinical Outcome

**DOI:** 10.3390/curroncol32020099

**Published:** 2025-02-10

**Authors:** Abhishek Mahajan, Ujjwal Agarwal, Renuka M. Ashtekar, Nivedita Chakrabarty, Richa Vaish, Vijay Maruti Patil, Vanita Noronha, Nandini Menon, Vasundhara Smriti, Jai Prakash Agarwal, Sarbani Ghosh-Laskar, Anil K. D’Cruz, Pankaj Chaturvedi, Prathamesh Pai, Asawari Patil, Munita Bal, Swapnil Rane, Neha Mittal, Kumar Prabhash

**Affiliations:** 1 Department of Imaging, The Clatterbridge Cancer Centre NHS Foundation Trust, Liverpool L7 8YA, UK; 2 Faculty of Health and Life Sciences, University of Liverpool, Liverpool L69 3BX, UK; 3 Department of Radiodiagnosis and Imaging, Tata Memorial Hospital, Homi Bhabha National Institute, Mumbai 400012, India; ujjwalagg8@gmail.com (U.A.); renukaashtekar1@gmail.com (R.M.A.); patilvr@tmc.gov.in (V.S.); 4 Tata Memorial Centre, Advanced Centre for Treatment, Research and Education in Cancer (ACTREC), Homi Bhabha National Institute (HBNI), Parel, Mumbai 400012, India; nchakrabarty@actrec.gov.in; 5 Department of Head and Neck Surgical Oncology, Tata Memorial Hospital, Homi Bhabha National Institute, Mumbai 400094, India; vaishr@tmc.gov.in (R.V.); dcruzak@tmc.gov.in (A.K.D.); director@actrec.gov.in (P.C.); drpspai@hncii.com (P.P.); 6 Department of Medical Oncology, Tata Memorial Hospital, Homi Bhabha National Institute, Mumbai 400094, India; vijaypgi@gmail.com (V.M.P.); noronhavm@tmc.gov.in (V.N.); menonns@tmc.gov.in (N.M.); prabhashk@tmc.gov.in (K.P.); 7 Department of Radiation Oncology, Tata Memorial Hospital, Homi Bhabha National Institute, Mumbai 400094, India; agarwaljp@tmc.gov.in (J.P.A.); laskarsg@tmc.gov.in (S.G.-L.); 8 Department of Pathology, Tata Memorial Hospital, Homi Bhabha National Institute, Mumbai 400094, India; apatil@actrec.gov.in (A.P.); balmm@actrec.gov.in (M.B.); srane@actrec.gov.in (S.R.); mittaln@tmc.gov.in (N.M.)

**Keywords:** HNSCC, oral cancer, CT, imaging, infratemporal fossa, AJCC 8th, TNM, HNSCC staging

## Abstract

Background: According to the 8th edition of the American Joint Committee on Cancer (AJCC), involvement of the masticator space and infratemporal fossa (ITF) in oral cancers indicates advanced disease (T4b), which is often considered unresectable. Previous studies have shown that the extent of ITF involvement influences management and outcomes. Therefore, to optimize management, T4b disease should be subclassified based on ITF involvement. Notably, infranotch disease has a more favorable prognosis compared to supranotch disease. Our study also observed that certain subsets of high anterior retroantral ITF involvement may be operable with favorable clinical outcomes. This study aims to derive a new image-based compartmentalization of high ITF involvement and assess its impact on the management and outcomes of oral head and neck squamous cell carcinoma (HNSCC) patients with high ITF involvement. Materials and Methods: This retrospective observational study included 154 non-metastatic, upfront unresectable locally advanced HNSCC patients who were fit for induction neoadjuvant chemotherapy (NACT). ITF involvement was classified into distinct compartments, and detailed staging of the primary tumor (T) and regional nodes (Ns) was performed. Clinical data, including patient demographics, treatment received, and follow-up notes, were documented. Prognosis was assessed using survival metrics: event-free survival (EFS), progression-free survival (PFS), and overall survival (OS). The ITF was categorized into the following compartments: compartment 1 (low ITF: medial pterygoid), compartment 2 (anterior high ITF: retroantral fat), compartment 3 (posterior high ITF), including 3a (paramandibular compartment: paramandibular fat/temporalis), 3b (muscle compartment: lateral pterygoid), and 3c (Perineural compartment: pterygopalatine fossa and pterygomaxillary fissure). Results: Of the 154 cases, 142 (92%) were classified as T4b, with 63 (40.9%) having high ITF involvement and 79 (55.6%) having low ITF involvement. Twelve cases had T4a disease, which was deemed unresectable due to extensive nodal involvement. Subcompartmentalization of the 63 high ITF cases revealed 26 (41.2%) with compartment 2 involvement, 17 (26.9%) with compartment 3a involvement, 11 (17.4%) with compartment 3b involvement, and 9 (14%) with compartment 3c involvement. Disease progression following NACT was significantly higher in compartment 3c, which showed a poor response (*p* = 0.007). Univariate analysis for PFS revealed similar outcomes for compartments 1 and 2 (*p* = 0.692), while compartment 3 demonstrated poorer outcomes (*p* = 0.033). Among thosehigh ITF involvement, compartment 3c had the worst PFS outcome (*p* = 0.03). Conclusions: Baseline imaging plays a critical role in guiding individualized treatment and predicting clinical outcomes. Low ITF involvement and disease limited to the high retroantral fat compartment exhibit similar clinical outcomes. Among the posterior high ITF compartments, involvement of the pterygopalatine fossa and pterygomaxillary fissure (compartment 3c) is associated with the worst prognosis and poor response to chemotherapy. Subcompartmentalization of ITF involvement provides valuable prognostic information to tailor treatment strategies.

## 1. Introduction

Buccal mucosa and gingivobuccal sulcus (GBS) squamous cell carcinoma (SCC) is one of the most prevalent oral cancers in India [1]. Many patients present with advanced-stage disease, leading to unfavorable outcomes and a high risk of local recurrence after treatment [2]. The 8th edition of the American Joint Committee on Cancer (AJCC) staging system, which uses contrast-enhanced computed tomography (CECT) as the primary diagnostic modality, classifies masticator space involvement as T4b, indicating advanced disease that is often considered unresectable [3,4]. Although masticator space involvement is typically associated with poor prognosis, it may not always preclude resection. We hypothesize that further subclassification of T4b disease based on clinical outcomes could offer more accurate prognostic markers, potentially guiding patient management more effectively and optimizing the use of limited healthcare resources [5].

**Figure 1:**
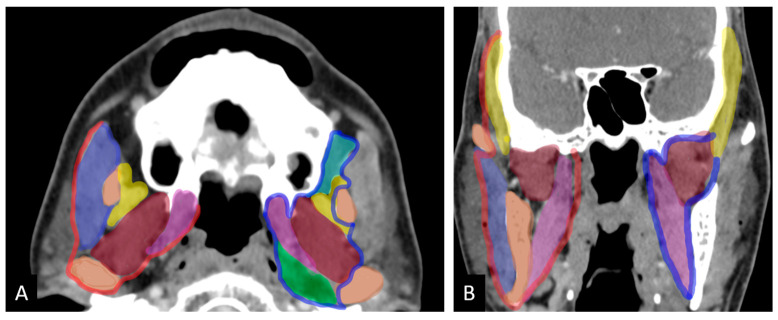
Axial (**A**) and coronal (**B**) images show masticator space on the right bounded by fascia (red) and infratemporal fossa bordered by an imaginary line (blue). Color code for muscles: purple: masseter; yellow: temporalis; brown: lateral pterygoid; pink: medial pterygoid; green: prestyloid parapharyangeal space; light green: retroantral space (adapted from Mahajan et al. ref [8]).

The masticator space is a suprahyoid deep fascial space that contains the ramus and posterior body of the mandible, the masseter, temporalis, medial, and lateral pterygoid muscles, and the mandibular division of the trigeminal nerve. It is formed by the splitting of the cervical fascia’s investing layer, with the inferior alveolar nerve exiting at the mandibular foramen [6,7]. The infratemporal fossa (ITF), which is bordered by the lateral pharyngeal wall, the ramus of the mandible, and the base of the skull, connects to the middle cranial fossa through the foramen ovale and spinosum, to the pterygopalatine fossa via the pterygomaxillary fissure, and to the temporal fossa via a deep gap in the zygomatic arch. Key components of the ITF include the pterygoid muscles, internal maxillary artery, pterygoid venous plexus, and mandibular division of the trigeminal nerve, but it does not contain the masseter (Figure 1) [7,8]. The medial portion of the masticator space, part of the parapharyngeal space, and the retroantral buccal space are included within the ITF.

Low ITF involvement (compartment 1) is generally considered “surgeon-friendly” and associated with favorable surgical outcomes. In contrast, high-ITF disease, or supra-notch disease, extends closer to the base of the skull and is typically linked to poor surgical outcomes and increased morbidity. This classification is widely accepted in clinical practice. However, we have observed that a specific subgroup of patients with high anterior retroantral ITF involvement (compartment 2) can undergo surgery with positive clinical outcomes [9,10]. These observations suggest that a more detailed imaging-based subclassification of T4b disease could better inform treatment decisions and improve patient care.

Advanced disease involving compartments 3a (temporalis muscle/paramandibular soft tissue), 3b (lateral pterygoid), and 3c (pterygopalatine fossa [PPF]/pterygomaxillary fissure [PMF]) can now be managed with neoadjuvant chemotherapy (NACT), potentially rendering these previously deemed inoperable cases resectable after tumor shrinkage. This evolving approach challenges prior assumptions that such cases were incurable. This hypothesis is supported by key trials, including TAX323 (Docetaxel + Cisplatin + 5-fluorouracil) and TAX324 (docetaxel, cisplatin, and 5FU vs. cisplatin and 5-fluorouracil), which demonstrated that induction chemotherapy is both safe and effective, enabling T4b patients to become candidates for resection [11,12,13].

**Figure 2:**
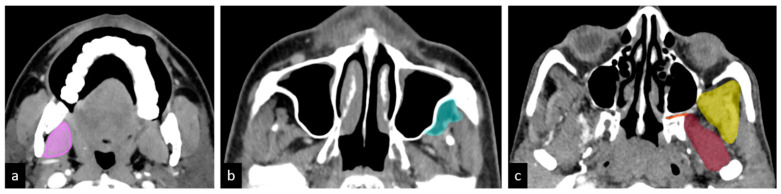
Hypothesized sub-compartments of the ITF: (**a**) compartment 1: low ITF—medial pterygoid (pink); (**b**) compartment 2: anterior high ITF—retroantral fat (light green); (**c**) compartment 3: posterior high ITF; compartment 3a: paramandibular compartment—paramandibular fat/temporalis (yellow); compartment 3b: muscle compartment—lateral pterygoid (brown); compartment 3c: perineural compartment—pterygopalatine fossa and pterygomaxillary fissure (orange) [8].

Given that the prognosis of infratemporal fossa (ITF) involvement varies based on the specific structures affected, we propose further subclassifying T4b disease into the following compartments (Figure 2):

- Compartment 1: Low ITF (medial pterygoid)
- Compartment 2: Anterior high ITF (retroantral fat)
- Compartment 3: Posterior high ITF
  ○ Compartment 3a: Paramandibular compartment (paramandibular fat/temporalis)
  ○ Compartment 3b: Muscle compartment (lateral pterygoid)
  ○ Compartment 3c: Perineural compartment (PPF/PMF).

We believe that incorporating this detailed compartmentalization into radiology reports will enhance communication between oncosurgeons and radiologists, improving decision-making, patient selection, and treatment planning. This approach could ultimately facilitate personalized cancer therapies, optimizing patient outcomes.

## 2. Materials and Methods

### 2.1. Study Population

**Figure 3:**
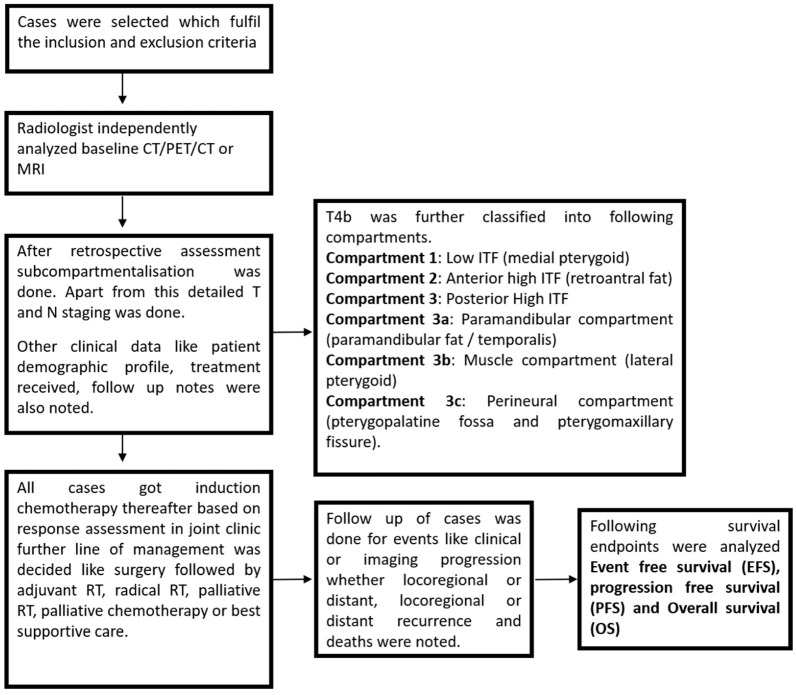
Flowchart for methodology.

This study cohort is a post hoc analysis of a phase III randomized controlled trial (CTRI/2016/04/006804) [14], conducted at a tertiary care cancer institute between 1 January 2016, and 31 December 2019. Patients were randomized 1:1 to receive either a three-drug combination (Cisplatin + 5-Fluorouracil + Docetaxel) or a two-drug regimen (Carboplatin + Docetaxel). Inclusion criteria comprised patients with primary, technically unresectable gingivobuccal cancer, who had pre-treatment baseline imaging (CT, PET-CT, or MRI) available on the Picture Archiving and Communication System (PACS) and treatment records. Exclusion criteria included cases with no available follow-up records, non-gingivobuccal cancer primary sites, or distant metastasis at presentation. Out of 494 cases screened, 344 were excluded due to missing baseline imaging or other primary malignancies (e.g., lip or skin). Thus, 154 cases were selected for analysis, as depicted in the flowchart (Figure 3).

### 2.2. Imaging Analysis

Baseline imaging (PET-CT, CT, or MRI) of the selected 154 cases was independently reviewed by a specialist head and neck radiologist with over 10 years of experience. The analysis focused on compartmentalization, followed by detailed primary tumor (T) and regional node (N) staging. Patient data, including age, sex, clinical findings, treatment received (chemotherapy, surgery, and radiotherapy), and response to treatment, were obtained from electronic medical records. Imaging was performed on reconstructed DICOM data using a volume viewer integrated within the PACS. The analysis included soft-tissue and bone algorithm reformation and axial images, employing triangulation for accurate compartmental assessment.

Compartmental Involvement Definitions:

- Compartment 1: Loss of fat planes with medial pterygoid.
- Compartment 2: Tumor extension into the high retroantral space, manifesting as mass or fat stranding.
- Compartment 3a: Loss of fat planes with temporalis muscle above the sigmoid notch or increased temporalis bulk with associated paramandibular soft tissue involvement.
- Compartment 3b: Loss of fat planes with lateral pterygoid muscle.
- Compartment 3c: Tumor extension into the pterygomaxillary fissure or pterygopalatine fossa.

T Staging: Detailed T staging was performed under the following categories:

- Epicenter of tumor;
- Size;
- Depth of invasion;
- Soft tissue extent: Retromolar trigone (RMT), gingivolingual sulcus, tongue, floor of mouth, masseter muscle, masticator space, medial pterygoid, low anterior retroantral fat, infratemporal fossa, extension into high infratemporal fossa, high anterior retroantral fat, temporalis muscle, lateral pterygoid, pterygomaxillary fissure, pterygopalatine fossa, condylar fossa, and intracranial extension.

Radiological Assessment of Bone and Perineural Spread:

- Cortical break adjacent to the tumor mass was considered malignant erosion.
- Contiguous trabecular destruction was defined as marrow invasion.
- Canal invasion: Tumor reaching into the canal with a breach of the bony canal wall, regarded as perineural spread along the inferior alveolar nerve.
- Perineural spread: Obliteration of fat or excessive enhancement within the mandibular foramen or foramen ovale, with or without widening or erosion of the foramen.

N Staging:

Detailed N staging included criteria for metastatic nodes such as round shape, loss of fatty hilum, necrosis, heterogeneous enhancement, and capsular irregularity. Extranodal extension (ENE) was identified by capsular irregularity with fat stranding or invasion, or gross muscle/vessel invasion.

### 2.3. Statistical Analysis

Statistical analysis was conducted using Stata (version 21) and SPSS (Statistical Package for the Social Sciences). Descriptive statistics were used for categorical variables, and comparisons were made using the chi-square test. Overall survival (OS) was calculated from the date of diagnosis to the date of death or the last follow-up, if applicable. Event-free survival (EFS) was calculated from diagnosis until the occurrence of a clinical or imaging event (local, regional, or distant progression, recurrence, or death), or until the last follow-up, if no event occurred. Progression-free survival (PFS) was calculated from diagnosis until disease progression, either clinically or on imaging, or until the last follow-up, if applicable. Survival outcomes were analyzed using Kaplan–Meier analysis, and comparisons were made using the log-rank test.

Additional covariates, including age at diagnosis, high vs. low ITF involvement, response to treatment, perineural spread, bone erosion, radiological skin involvement, radiological extranodal status, nodal necrosis, and surgery vs. non-surgery, were also tested for prognostic significance. Statistically significant and clinically relevant covariates were included in a multivariate Cox proportional hazards regression model. Survival curves were generated using RStudio (version 1.2.1335) by the Kaplan–Meier method to demonstrate the effect of compartmentalization on progression and survival. A chi-square test was used to assess the association between sub-compartments and response to neoadjuvant chemotherapy (NACT).

## 3. Results

### 3.1. Patient Characteristics

Among the 154 cases, the majority of patients were male (90.3%), with a mean age at diagnosis of 44 years. Twelve patients were excluded from the analysis due to stage T4a disease, which was deemed unresectable due to extensive nodal involvement. The remaining 142 patients (92%) had locally advanced T4b disease, of which 40.9% exhibited high ITF involvement on imaging, and 55.6% had low ITF involvement (compartment 1). Additionally, 42.9% of patients were categorized as N3b, while the remaining 47.1% had other N stages. The patients’ characteristics are summarized in Table 1.

### 3.2. Subcompartmentalization and Impact on Response to NACT

Upon further subcompartmentalization of high ITF involvement, 41.2% of cases exhibited compartment 2 involvement, 26.9% had compartment 3a involvement, 17.4% had compartment 3b involvement, and 14% had compartment 3c involvement. After completing two cycles of neoadjuvant chemotherapy (NACT), cases were evaluated in a joint clinic and categorized based on the latest version of the Response Evaluation Criteria in Solid Tumours (RECIST 1.1) into partial response (PR), stable disease (SD), and progressive disease (PD) according to clinical and imaging findings.

Out of 154 cases, a response assessment was performed for 150 patients (2 patients were excluded due to chemotoxicity, and 2 patients expired during NACT). Among the 150 patients, 56% had SD, 24.6% had PR, and 19.3% had PD. Of the 56% with SD, 45.2% had compartment 1 involvement, 18.6% had compartment 2 involvement, 11.6% had compartment 3a involvement, 9.5% had compartment 3b involvement, and 4.6% had compartment 3c involvement. Among the 24.6% with PR, 62.1% had compartment 1 involvement, 18.9% had compartment 2 involvement, 10.8% had compartment 3a involvement, 2% had compartment 3b involvement, and none had compartment 3c involvement. Of the 19.3% with PD, 58.6% had compartment 1 involvement, 6.8% had compartment 2 involvement, 10.3% had compartment 3a involvement, 6.8% had compartment 3b involvement, and 17.2% had compartment 3c involvement. Table 2 summarizes the compartment-wise response to NACT and surgical resection.

### 3.3. Subcompartmentalization and Impact on Management

Following neoadjuvant chemotherapy (NACT), 61 patients underwent surgery. Of these, 60.7% had compartment 1 involvement, 18% had compartment 2 involvement, 8.2% had compartment 3a involvement, 4.9% had compartment 3b involvement, and none had compartment 3c involvement. Additionally, 8.1% of cases were deemed unresectable due to extensive nodal involvement (T4a). All 61 patients received adjuvant radiation (60 Gray/30 fractions over 6 weeks), and 45 patients received concurrent chemotherapy.

Chi-square cross-tabulation revealed a significant association between high ITF subcompartments and response to NACT (*p* = 0.019). Compartment 3c involvement was significantly associated with poor response, evidenced by disease progression (*p* = 0.007), while compartment 2 involvement showed a favorable response with partial response (PR) or stable disease (SD) (*p* = 0.047) (Table 3). Notably, 46% of patients with SD according to RECIST 1.1 underwent resection, compared to 57.1% of patients with PR, highlighting a potential limitation of using the RECIST 1.1 criteria for head and neck cancers.

Histopathological analysis of the 61 cases revealed that 22.9% were poorly differentiated, while 77% were moderately or well-differentiated. All patients had negative surgical margins. Additionally, 19.6% exhibited histological perineural invasion, 6.5% had lymphovascular invasion, and 40.9% showed positive extranodal extension. Table 4 summarizes compartment-wise management following NACT and surgical resection.

### 3.4. Subcompartmentalization and Impact on Progression Free Survival

**Figure 4:**
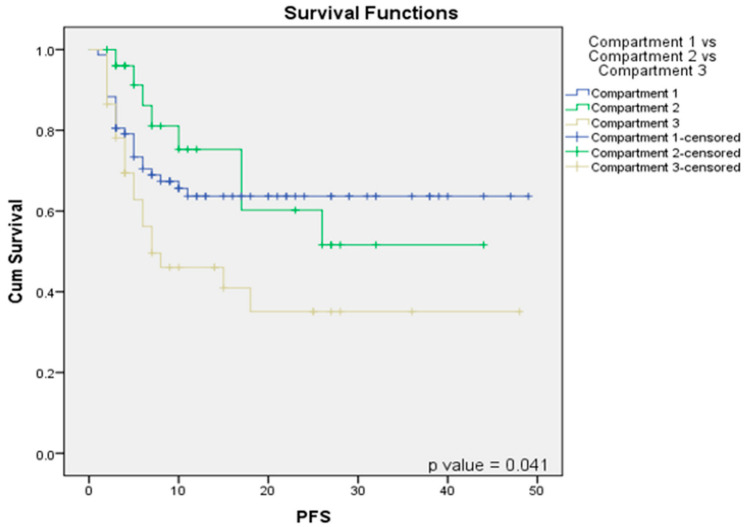
Survival function Kaplan–Meier curves correlating compartment 1 vs. compartment 2 vs. compartment 3 with PFS.

Our hypothesis posited that high ITF sub-compartment involvement results in different clinical outcomes, with compartment 1 (low ITF) and compartment 2 (limited to anterior high ITF retroantral fat) behaving similarly. Survival analysis for progression-free survival (PFS) revealed no significant difference between compartment 1 and compartment 2, with a *p*-value of 0.685, indicating comparable PFS outcomes for both compartments (Figure 4) (Table 5).

However, when comparing PFS among compartment 1 (low ITF), compartment 2 (anterior high ITF), and compartment 3 (posterior high ITF), a significantly poorer PFS was observed for compartment 3 (*p* = 0.041), as compared to compartments 1 and 2 (Figure 4). Within the high-ITF-involvement patients, compartment 3a (*p* = 0.05) and compartment 3c (*p* = 0.03) demonstrated significantly worse PFS outcomes than compartment 3b (Table 5).

Additionally, radiological evidence of extranodal extension (ENE) was associated with significantly poorer event-free and progression-free outcomes compared to ENE-negative cases. Response to NACT and subsequent surgery also significantly impacted PFS, event-free survival, and overall survival.

## 4. Discussion

The involvement of the masticator space and infratemporal fossa (ITF) in oral cancers is generally associated with poor prognosis. However, our study suggests that this correlation may not always hold true, and proposes a subcompartmentalization of the ITF based on distinct clinical outcomes. This approach could aid in more accurate prognostication and tailored management strategies.

Our cohort included 154 cases, with 142 (92%) at the locally advanced T4b stage. Of these, 63 (40.9%) had high ITF involvement, while 79 (55.6%) exhibited low ITF involvement. Nodal involvement was also varied, with 66 cases (42.9%) categorized as N3b, and 88 cases (57.1%) in other N stages. Among the 63 high ITF cases, 26 (41.2%) were classified into compartment 2, 17 (26.9%) into compartment 3a, 11 (17.4%) into compartment 3b, and 9 (14%) into compartment 3c.

Neoadjuvant chemotherapy (NACT) was commonly administered for reasons such as edema extending to the zygoma, high ITF involvement, carotid abutment, and mandibular preservation. Among the 77 cases in compartment 1, 37 (48%) underwent resection post-NACT, with all receiving adjuvant radiotherapy (RT) and some also undergoing concurrent chemotherapy. Similar patterns were observed across compartments 2, 3a, 3b, and 3c, with varying rates of post-NACT resection and adjuvant treatments. Chi-square analysis revealed significant correlations between ITF sub-compartments and NACT response. Notably, compartment 3c demonstrated the poorest response and disease progression, while compartments 1 and 2 exhibited better and comparable responses.

Previous studies have attempted a subclassification of the ITF to predict prognosis and resectability in head and neck cancers. Liao et al. [15,16] introduced the concept of the sigmoid notch, suggesting that disease extending above the notch had poorer outcomes compared to infranotch involvement. However, in our experience, disease limited to the retroantral fat with high ITF involvement had similar outcomes to low ITF involvement. Poorer outcomes were observed only when other supranotch structures, such as the temporalis muscle, lateral pterygoid muscle, and pterygomaxillary fossa (PMF)/pterygopalatine fossa (PPF), were involved. Similarly, Mohiyuddin et al. [17] reported worse outcomes for tumors extending into the lateral pterygoid (supranotch) compared to infranotch disease. Trivedi et al. [10] evaluated the pathological extent of disease through compartment resection, regardless of the extent of involvement, and assessed margin control and clinical outcomes. Kumar et al. [18] found that survival rates in T4b oral cancers with the involvement of three or fewer masticatory space structures were comparable to those with T4a.

**Figure 5:**
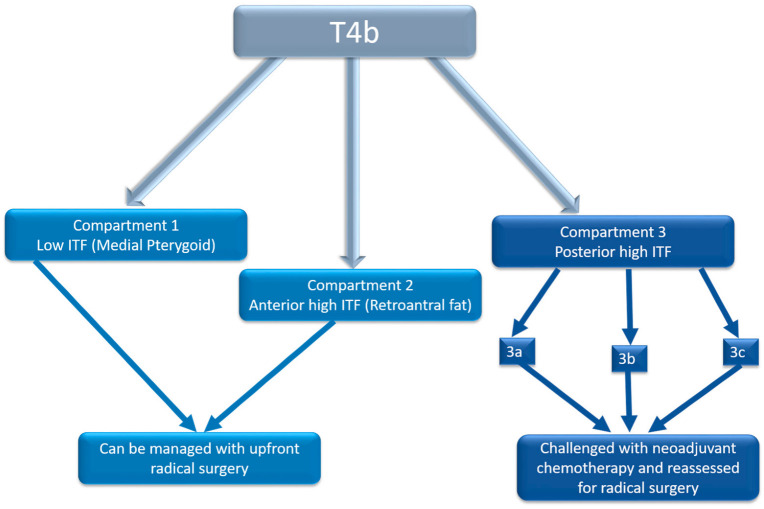
Flowchart demonstrating bioselection on baseline imaging for guiding treatment.

Based on our findings, we recommend using baseline imaging to predict clinical outcomes based on ITF sub-compartments. Specifically, disease confined to the high retroantral fat should be considered part of the low ITF compartment, as it behaves similarly. Posterior high ITF involvement (compartment 3) is associated with significantly worse outcomes compared to compartments 1 and 2. Among posterior high ITF compartments, the involvement of 3c and 3a carries significantly worse prognoses compared to compartment 3b (Figure 5).

**Prognostic Implications of Compartments 3a and 3c**

The poor prognosis associated with compartments 3a and 3c can be attributed to several anatomical and pathological factors. Compartment 3a, which includes the temporalis muscle and adjacent paramandibular fat, serves as a conduit for tumor invasion toward the skull base. A tumor spreading into this region often results in extensive soft tissue infiltration, complicating surgical resection and increasing the likelihood of residual disease.

Compartment 3c, encompassing the pterygopalatine fossa (PPF) and pterygomaxillary fissure (PMF), is particularly concerning due to its role in perineural tumor spread along branches of the trigeminal nerve. This region is critical for intracranial extension, contributing to poor outcomes. Perineural invasion (PNI) has been consistently linked to poor responses to chemotherapy and radiotherapy, leading to high recurrence rates.

**Anatomical Considerations and Treatment Challenges**

The anatomical complexity of compartments 3a and 3c poses significant challenges in both surgical and non-surgical management. The proximity of compartment 3c to vital neurovascular structures limits the possibility of complete tumor resection without risking critical functions. Perineural spread within this compartment often necessitates aggressive multimodal therapy, including chemoradiation, to achieve adequate disease control.

In compartment 3a, the involvement of the temporalis muscle and adjacent structures increases the risk of residual disease following surgery. Additionally, the paramandibular soft tissue serves as a conduit for tumor dissemination, further complicating treatment strategies.

**Advances in Imaging and Future Directions**

Recent advancements in imaging, particularly MRI and image fusion techniques, have significantly improved the evaluation of cranial nerve involvement and tumor extension in high-risk compartments. The integration of MRI with multi-detector computed tomography (MDCT) in radiation therapy planning enhances tumor margin delineation and better visualizes perineural invasion, facilitating more precise treatment delivery.

Studies have demonstrated the utility of advanced MRI techniques, such as 3D cranial nerve imaging and black-blood STIR TSE sequences, in visualizing extraforaminal cranial nerve branches and assessing tumor spread along neural pathways [19,20,21,22]. These modalities provide essential information for surgical decision-making, especially in determining resectability and the need for adjunctive therapies.

Moreover, MRI fusion with MDCT radiation therapy planning has emerged as a promising approach for optimizing radiation delivery in cases with extensive perineural invasion. By integrating high-resolution MRI data with radiation planning software, clinicians can achieve more accurate target delineation, minimizing radiation exposure to surrounding normal tissues while ensuring adequate tumor coverage [20,21,23].

**Decision-Making Regarding Cranial Nerve Involvement**

The involvement of the mandibular division of the trigeminal nerve (V3) in compartments 3a and 3c presents a significant challenge in treatment planning. Extensive perineural spread along V3 is often associated with poor prognosis and an increased risk of distant metastases. Early detection of perineural invasion using advanced imaging techniques is crucial, as it influences decisions regarding surgical resectability and the need for intensified adjuvant therapy.

Recent studies have highlighted the role of 3D CRANI [24] in reliably visualizing extraforaminal cranial and occipital nerves, offering new insights into tumor-nerve interactions. This has important implications for surgical planning, particularly in cases where nerve preservation is a key consideration.

The poor prognosis associated with compartments 3a and 3c emphasizes the need for precise imaging, tailored treatment strategies, and continued research into advanced imaging techniques. The integration of MRI fusion with MDCT in radiation therapy planning represents a significant advancement in optimizing treatment outcomes for patients with high-risk ITF involvement. Future studies should focus on refining imaging protocols and developing novel therapeutic approaches to improve prognosis in these challenging cases.

## 5. Conclusions

Baseline imaging plays a crucial role in patient selection, guiding personalized treatment strategies, and predicting clinical outcomes. We recommend incorporating the high retroantral fat compartment into the low ITF compartment within the AJCC staging system, as both regions exhibit similar clinical behavior. Among the high ITF sub-compartments, 3a and 3c are associated with the poorest prognosis and response to chemotherapy, while compartment 3b demonstrates relatively better outcomes, although still inferior to compartment 2. This compartmentalization of the ITF correlates with response to neoadjuvant chemotherapy (NACT), underscoring its value in treatment planning. Such bioselection based on imaging findings is essential for optimizing therapeutic approaches and improving patient outcomes.

## Figures and Tables

**Table 1:** Patient characteristics.

Patient Characteristics		
Clinical Variables	Number (n)	Percentage (%)
**1. Gender**		
Male	139	90.3
Female	15	9.7
**2. Age (years, median)**		
Age > 40 years	104	67.5
Age < 40 years	50	32.5
**3. Sites of primary tumor**		
Right buccal mucosa	71	46.1
Left buccal mucosa	70	45.5
Alveolus	11	7.1
Retromolar trigone	2	1.3
**4. T stage**		
Low ITF (Compartment 1)	79	55.6
High ITF	63	40.9
Compartment 2	26	41.2
Compartment 3a	17	26.9
Compartment 3b	11	17.4
Compartment 3c	9	14
**5. rDepth of invasion (rDOI)**		
More than 10 mm	92	59.7
Less than 10 mm	62	40.3
**6. rPerineural spread(rPNI)**		
Yes	41	26.6
No	113	73.4
**7 Radiological skin involvement**		
Yes	58	37.7
No	96	62.3
**8. Radiological nodal metastasis**		
rN3b	66	42.9
rOther N stages	88	47.1
**9. Chemotherapy arm**		
DC-arm	82	53.2
DCF-arm	72	46.8
**10. Response**		
Stable disease	84	56
Partial response	37	24.6
Progressive disease	29	19.3
**11. Surgery**		
Yes	61	40.9
No	89	59.7
**12. Histopathological Characteristics**		
Margins negative	61	100
Perineural invasion	12	19.6
Lymphovascular invasion	04	6.5
Positive nodes	35	57.3
Extracapsular extension	25	40.9
Poor grade	14	22.9
**13. Radiotherapy received**	83	55.3
Adjuvant	61	73.4
Definitive	7	8.4
Palliative	15	18
**14. Palliative chemotherapy received**	55	36.6
**15. Progression**	58	37.6
Locoregional	55	94.8
Distant	3	5.2
**16. Deaths**	29	18.8

r: radiologic. ITF: infratemporal fossa.

**Table 2:** Compartment-wise response to NACT and surgical resection.

Compartment Wise Response to NACT and Surgical Resection					
Compartment	Total Cases	SD	PR	PD	Surgery
**Compartment 1**	77	38 (49.3%)	23 (29.8%)	17 (22%)	37 (48%)
**Compartment 2**	26	16 (61.5%)	7 (26.9%)	2 (7.6%)	11 (42.3%)
**Compartment 3a**	17	10 (58.8%)	4 (23.5%)	3 (17.6%)	5 (29.4%)
**Compartment 3b**	11	8 (72.7%)	1 (9%)	2 (18.1%)	3 (27.2%)
**Compartment 3c**	9	4 (44.4%)	0 (0%)	5 (55.5%)	0 (0%)

SD: stable disease; PR: partial response; PD: progressive disease.

**Table 3:** Cross-tabulation showing the association between sub-compartments (high ITF) and response to NACT.

Compartment	Response to NACT		Total	*p*-Value
	Stable Response and Partial Response	Disease Progression		
**Compartment 2**	92.3% (24)	7.7% (2)	100% (26)	0.047
**Compartment 3a**	82.4% (14)	17.6% (3)	100% (17)	0.332
**Compartment 3b**	81.8% (9)	18.2% (2)	100% (11)	0.361
**Compartment 3c**	44.4% (4)	55.6% (5)	100% (9)	0.007
**Total**	81% (51)	19% (12)	100% (63)	

**Table 4:** Compartment-wise management post NACT +/− surgical resection.

Compartment Wise Management Post NACT					
Compartment	Total Cases	Surgery + Adjuvant RT	Definitive RT	Palliative RT	Palliative Chemo
Compartment 1	77	37 (48%)	2 (2.5%)	8 (10.3%)	25 (32.4%)
Compartment 2	26	11 (42.3%)	2 (7.6%)	1 (3.8%)	11 (42.3%)
Compartment 3a	17	5 (29.4%)	0 (0%)	2 (11.7%)	6 (35.2%)
Compartment 3b	11	3 (27.2%)	2 (18.1%)	2 (18.1%)	2(18.1%)
Compartment 3c	9	0 (0%)	1 (11.1%)	1 (11.1%)	7 (77.7%)

RT: radiation therapy, NACT: neoadjuvant chemotherapy.

**Table 5:** Association between sub-compartments and PFS.

Progression Free Survival (Compartment Wise)					
		HR	95.0% CI for Exp(B)		*p* Value
			Lower	Upper	
Compartment 1 vs. Compartment 2	Compartment 1				
	Compartment 2	0.852	0.386	1.882	0.685
Compartment 1 vs. Compartment 2 vs. Compartment 3	Compartment 1				
	Compartment 2	0.854	0.387	1.886	0.696
	Compartment 3	1.888	1.052	3.388	0.033
Compartment 2 vs. Compartment 3	Compartment 2				
	Compartment 3	2.368	1.038	5.402	0.040

## Data Availability

The original contributions presented in the study are included in the article. Further inquiries can be directed to the corresponding author.
